# Supplementary material for: Surface hydrophobization of zeolite enables mass transfer matching in gas-liquid-solid three-phase hydrogenation under ambient pressure
Source: Nat Commun. 2024 Mar 7;15:2076. doi: 10.1038/s41467-024-46505-3 (PMC10920826; doi:10.1038/s41467-024-46505-3)
Supplement: Supplementary file 1 — Supplementary information [file 41467_2024_46505_MOESM1_ESM.pdf]

Supplementary Information

Surface hydrophobization of zeolite enables mass transfer matching in gas-liquid-solid three-phase hydrogenation under ambient pressure

Shuai Wang,[1] Riming Hu,[2] Jianyu Ren,[3] Yipin Lv,[1] Lianghao Song,[4] Huaiqing Zhao,[1] Xuchuan Jiang,[2] Daowei Gao[1]✉ & Guozhu Chen[1]✉

1、 School of Chemistry and Chemical Engineering, University of Jinan, Jinan 250022, PR China

2、 Institute for Smart Materials & Engineering, University of Jinan, Jinan 250022, PR China

3、 Department of Chemistry and Biochemistry, University of California San Diego, La Jolla CA 92093, USA

4、 Department of Chemistry, Sungkyunkwan University, Suwon 16419, Korea

Correspondence and requests for materials should be addressed to D.G. (email: chm_gaodw@ujn.edu.cn) or to G.C. (email: chm_chengz@ujn.edu.cn).

Catalyst characterization

X-ray photoelectron spectroscopy (XPS) was performed on a PHI5000 Versaprobe System. The X-ray absorption spectroscopy (XAS) including X-ray absorption near-edge structure (XANES) and extended X-ray absorption fine structure (EXAFS) spectra at the Pt L_3-edge were collected in BL14W1 station in Shanghai Synchrotron Radiation Facility (SSRF) (Shanghai, China). Before the analysis at the beamline, samples were pressed into thin sheets with 1 cm in diameter and sealed using Kapton tape film. The XAFS spectra were recorded at room temperature using a 4-channel Silicon Drift Detector (SDD) Bruker 5040. Pt *L*-edge EXAFS spectra were recorded in transmission mode. Negligible changes in the line-shape and peak position of Pt *L*-edge XANES spectra were observed between two scans taken for a specific sample. The XAFS spectra of these standard samples (Pt foil and PtO_2) were recorded in transmission mode. The spectra were processed and analyzed by the software codes Athena and Artemis.

Adsorption experiments

In typical procedure, the adsorption experiment was carried out in a 15 mL glass bottle containing 5 mL water solution with 0.47 mmol benzaldehyde (or benzyl alcohol). Benzaldehyde (or benzyl alcohol) was dispersed evenly in water by ultrasound, and then as-prepared catalyst was added. The glass bottle equipped with magnetic stirring (900rpm) was heated to 50 ºC, and the adsorption experiment lasted for 1h. Subsequently, the catalyst and the mixture were separated by filtration. The aqueous solution was extracted with ethyl acetate to ensure that the unadsorbed benzaldehyde (or benzyl alcohol) is dissolved into the ethyl acetate. The catalyst was extracted using

ethyl acetate, guaranteeing the sufficiently desorption of benzaldehyde (or benzyl alcohol) adsorbed on the catalyst into the ethyl acetate phase. After that, quantitative analyses were conducted by GC. The dosage of benzaldehyde (or benzyl alcohol) adsorption was calculated out by the difference of concentration before and after the adsorption experiment. The carbon balance of the adsorption experiments approximated 95~97%.

Molecular dynamics (MD) simulation methods and models

The simulations are utilized to gain insights into the diffusion rates of molecules. For this purpose, an all-atomic molecular dynamics simulation model was employed to focus on the molecular level details. LAMMPS simulation package was used for all MD simulations.[1] The structure of HieTS-1 was obtained by randomly replacing a third of Si atoms with Ti atoms in S-1.[2,3] The structure of TS-1-C_3 was obtained by modifying the HieTS-1 structure and the unsaturated O atoms were bonded with additional trimethoxy (propyl) silane to remain the framework stable. Both the HieTS-1 and HieTS-1-C_3 models were optimized using the simulated annealing (SA) method with temperature cooled down from 500 to 300 K for three times and a 200 ps MD simulation. No geometry collapse was observed after SA and MD simulations, proving that the constructed structures possess a very strong stability. The dimensions of HieTS-1 and HieTS-1-C_3 cubes were both 80.43×80.43×80.43, and all the atom positions were fixed. The benzaldehyde molecule, water molecule and hydrogen molecule were optimized by density function theory (DFT) using VASP (version 6.3.0).[4-6] 400 molecules of benzaldehyde molecules and 2640 molecules of water molecules (solvent) were put on

the left of HieTS-1 and HieTS-1-C_3 with the distance about 0.5 nm, separately. Furthermore, 400 molecules of hydrogen molecules and 2640 molecules of water molecules (solvent) were put on the left of HieTS-1 and HieTS-1-C_3 with the distance about 0.5 nm, separately. All MD simulations were performed using the NVT ensemble. The temperature was maintained at 300K using the Berendsen temperature coupling method[7]. The simulations were performed over 1 ns. All the frames of trajectory were extracted and analyzed to check the diffusion rates of molecules. The mobilities of benzaldehyde molecules and hydrogen molecules upon diffusion in HieTS-1 and HieTS-1-C_3 were examined by mean-squared displacement (MSD), respectively:

$$\mathrm{MSD(t)} = \frac{1}{N}\sum_{i=1}^{N} < |\mathrm{ri}(t) - \mathrm{ri}(0)|^2 > \qquad (1)$$

where N is the number of benzaldehyde molecules or hydrogen molecules and ri(t) is the position of the it(h) benzaldehyde molecule or hydrogen molecule at time t.

Density functional theory (DFT) calculations computational details

All the spin-polarized density functional theory calculations were performed by using the Vienna ab initio Simulation Program (VASP). The structure of TS-1 was obtained by randomly replacing a third of Si atoms with Ti atoms in S-1.[2,3] The generalized gradient approximation (GGA) in the Perdew-Burke-Ernzerhof (PBE) form and a cutoff energy of 500 eV for planewave basis set were adopted. A 3 × 3 × 1 Monkhorst-Pack grid was used for sampling the Brillouin zones at structure optimization. The ion-electron interactions were described by the projector augmented wave (PAW) method. A Gaussian smearing of 0.1 eV to the orbital occupation is applied during the geometry optimization and for the total energy computations. The

convergence criteria of structure optimization were chosen as the maximum force on each atom less than 0.02 eV/Ǻ with an energy change less than 1×10^{-5} eV. The DFT-D3 semiempirical correction was described via Grimme's scheme method. To calculate the kinetic energy barrier of chemical reactions, the climbing image nudged elastic band (CI-NEB) method was used to search for the transition states.

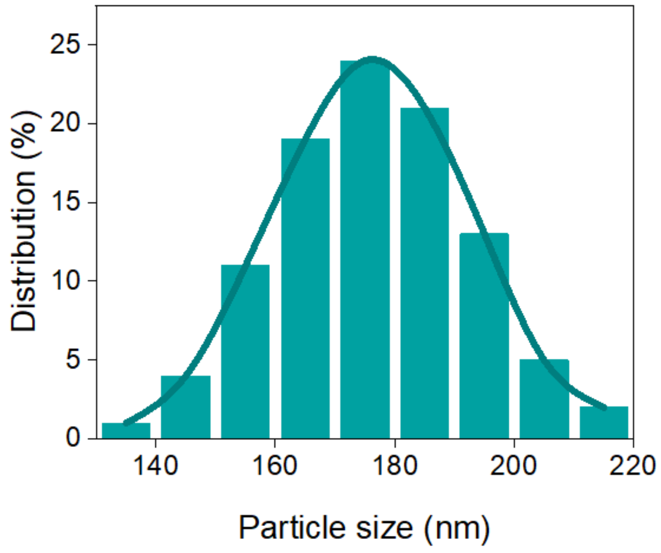

Supplementary Figure 1 Particle size distribution of Pt@HieTS-1.

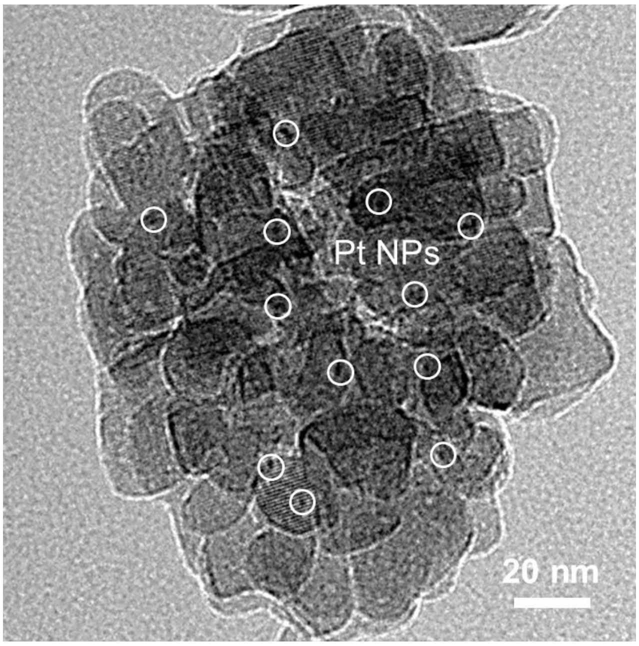

Supplementary Figure 2 HRTEM image of Pt@HieTS-1 zeolite.

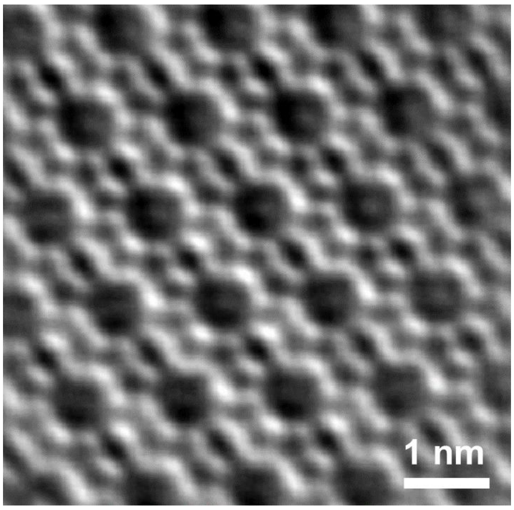

Supplementary Figure 3 High-magnification Cs-corrected iDPC STEM image of Pt@HieTS-1 zeolite.

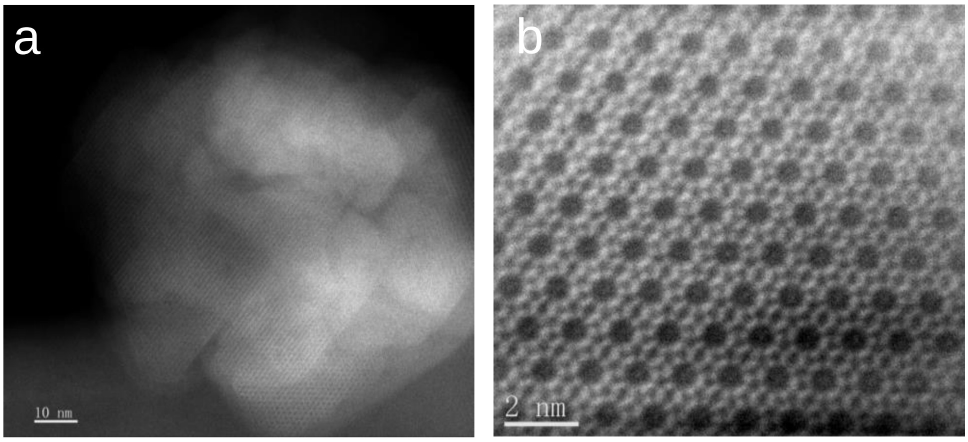

Supplementary Figure 4 (a) Cs-corrected HAADF STEM image of HieTS-1 zeolite.

(c) Cs-corrected iDPC STEM image of HieTS-1 zeolite.

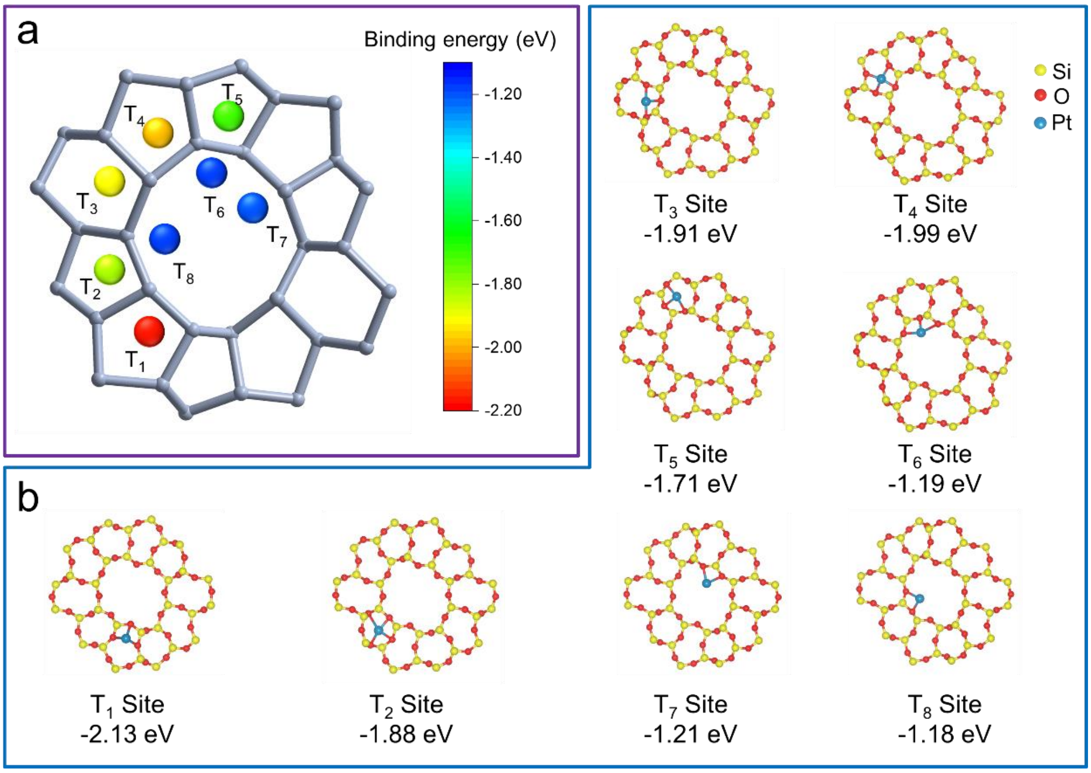

Supplementary Figure 5 Optimized structure of atomically dispersed Pt species on HieTS-1 by DFT calculations with binding energy shown in eV.

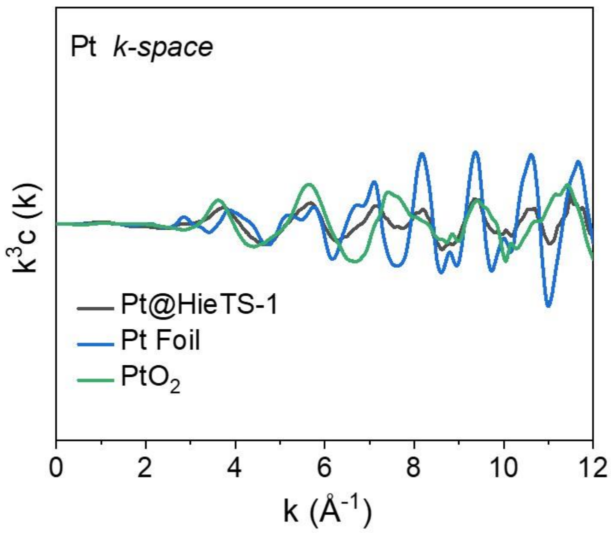

Supplementary Figure 6 *K*-spaced FT EXAFS spectra of Pt@HieTS-1, Pt foil and PtO_2.

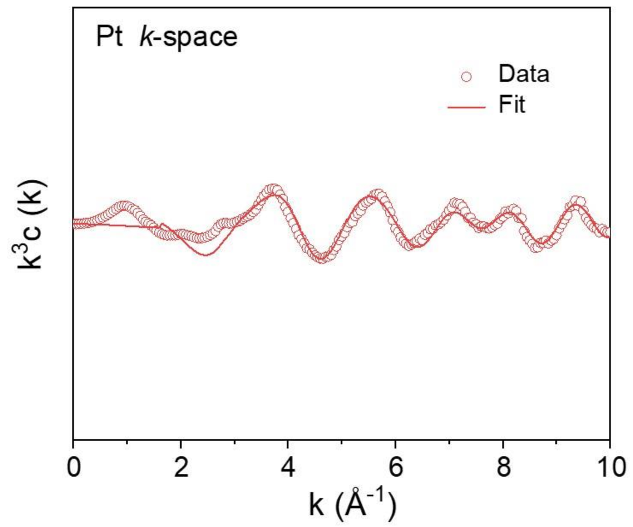

Supplementary Figure 7 FT EXAFS fitting spectrum of Pt@ HieTS-1 at *K* space.

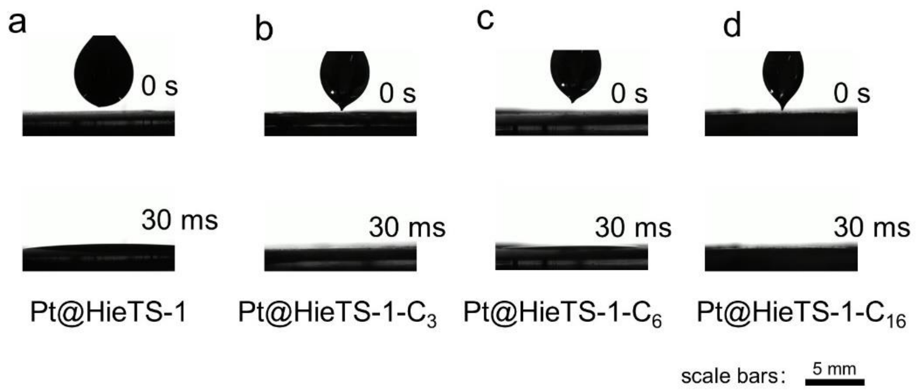

Supplementary Figure 8 Contact angles of benzaldehyde with (a) Pt@HieTS-1, (b)Pt@HieTS-1-C_3, (c) Pt@HieTS-1-C_6 and (d) Pt@HieTS-1-C_{16}.

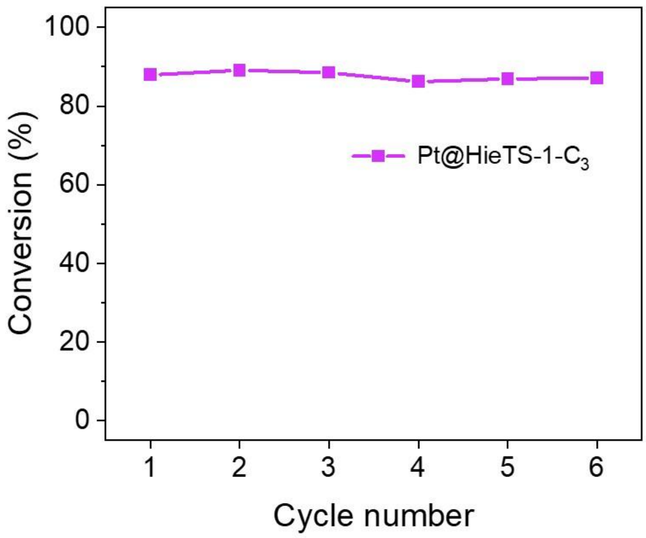

Supplementary Figure 9 Recycling results of benzaldehyde hydrogenation over Pt@HieTS-1-C_3.

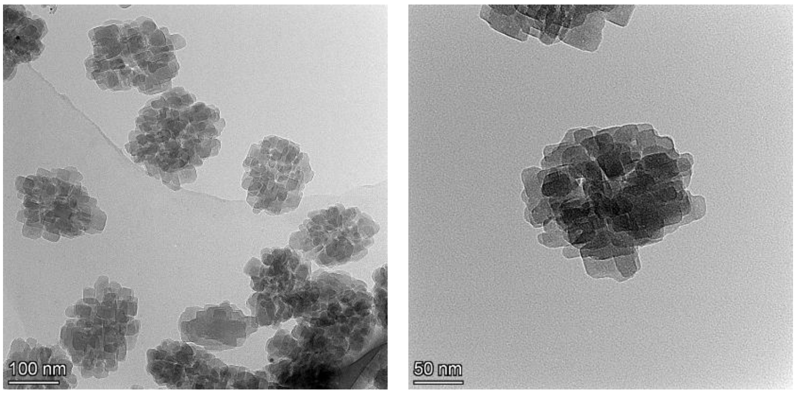

Supplementary Figure 10 TEM images of Pt@HieTS-1-C_3 after the reaction.

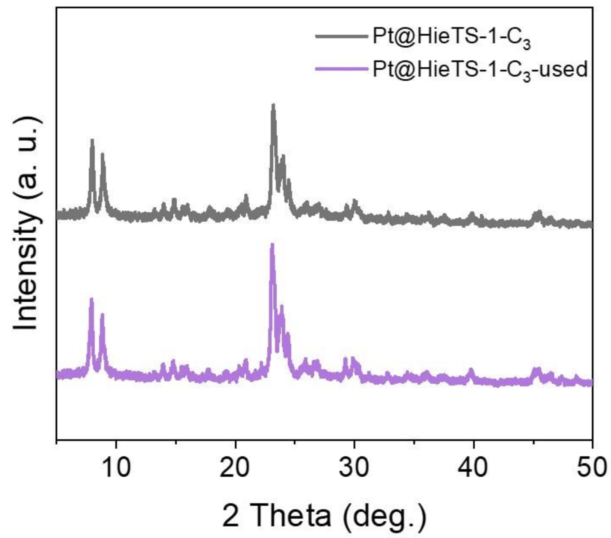

Supplementary Figure 11 XRD patterns of Pt@HieTS-1-C_3 after the reaction.

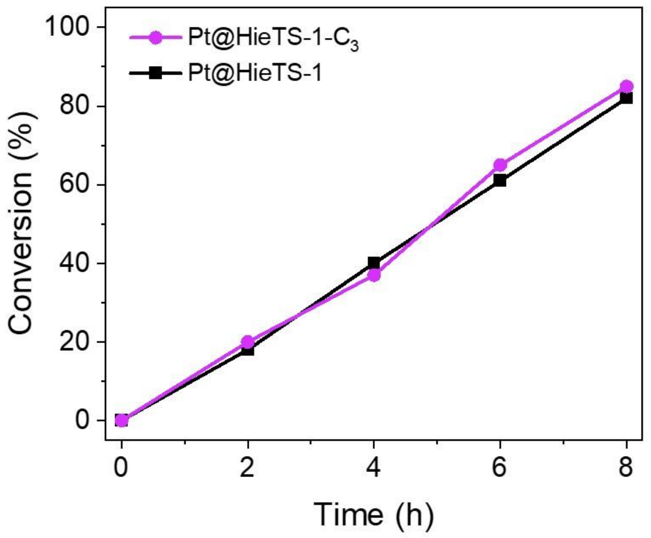

Supplementary Figure 12 The evolution of benzaldehyde conversion in ethanol versus the reaction time for Pt@HieTS-1-C_3 and Pt@HieTS-1.

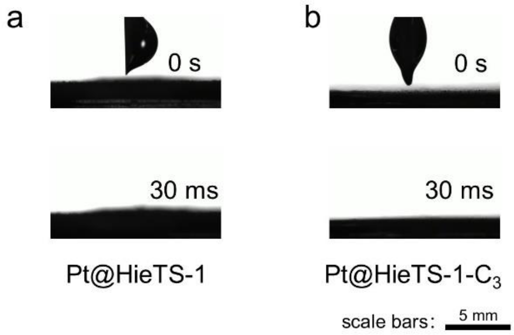

Supplementary Figure 13 Contact angles of ethanol with (a) Pt@HieTS-1 and (b) Pt@HieTS-1-C_3.

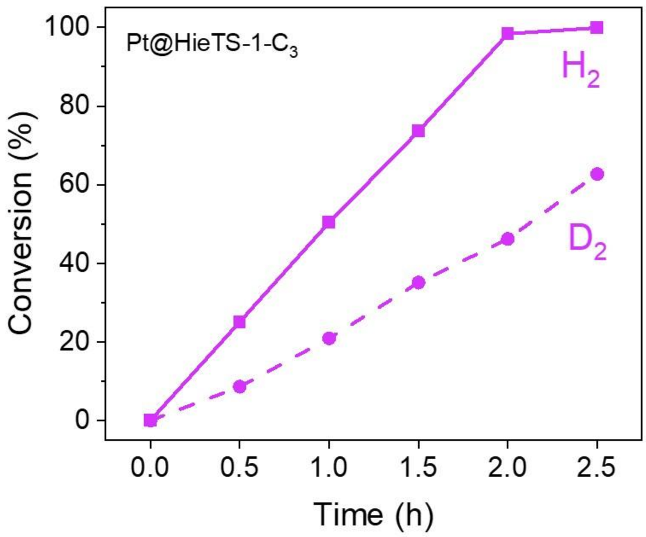

Supplementary Figure 14 Kinetic isotope effect of H_2/D_2 on benzaldehyde hydrogenation.

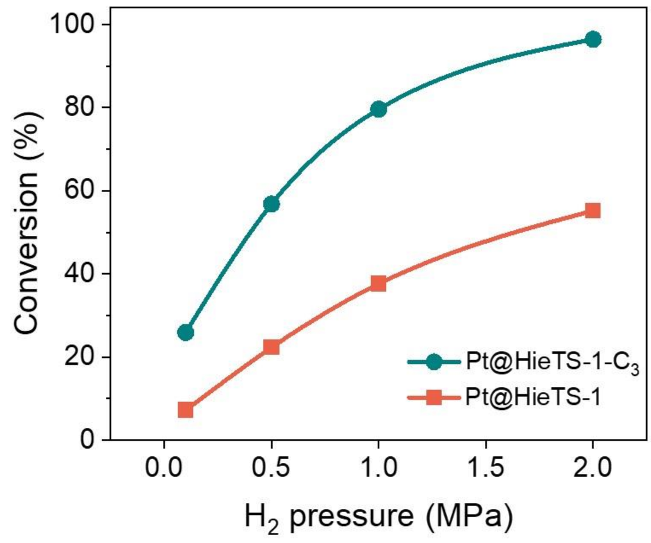

Supplementary Figure 15 The effect of the H_2 pressure on benzaldehyde hydrogenation. Reaction condition: benzaldehyde (0.47 mmol), Pt dosage (2.1×10^{-3} mmol), water (5 mL), 50 ºC, 0.5 h.

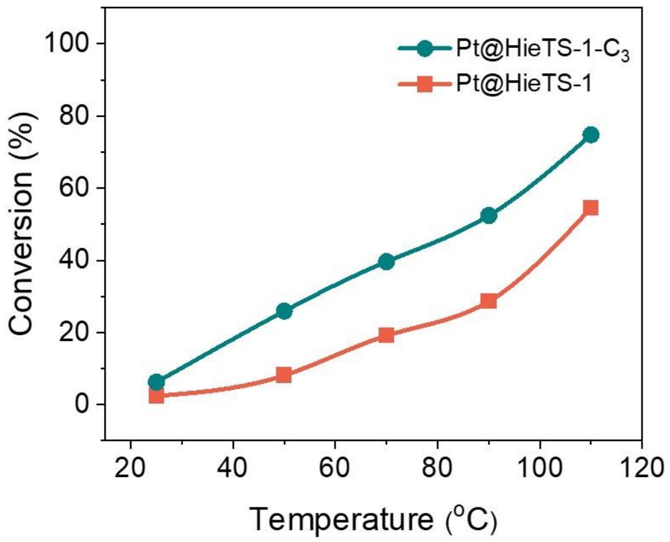

Supplementary Figure 16 The effect of the reaction temperature on benzaldehyde hydrogenation. Reaction condition: benzaldehyde (0.47 mmol), Pt dosage (2.1×10^{-3} mmol), water (5 mL), 1 atm H_2, 0.5 h.

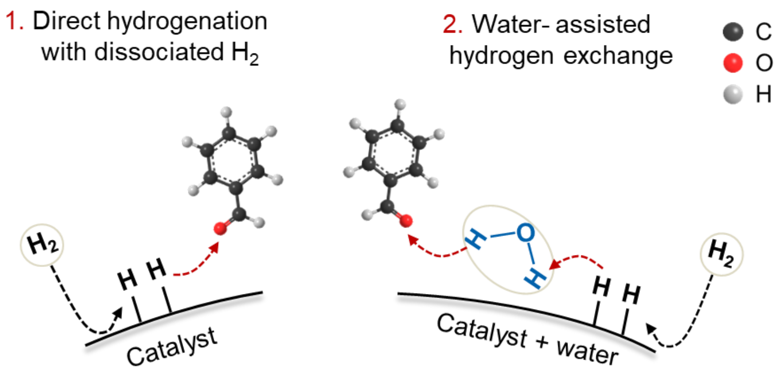

Supplementary Figure 17 The schematic diagram of two possible reaction pathways for benzaldehyde hydrogenation.

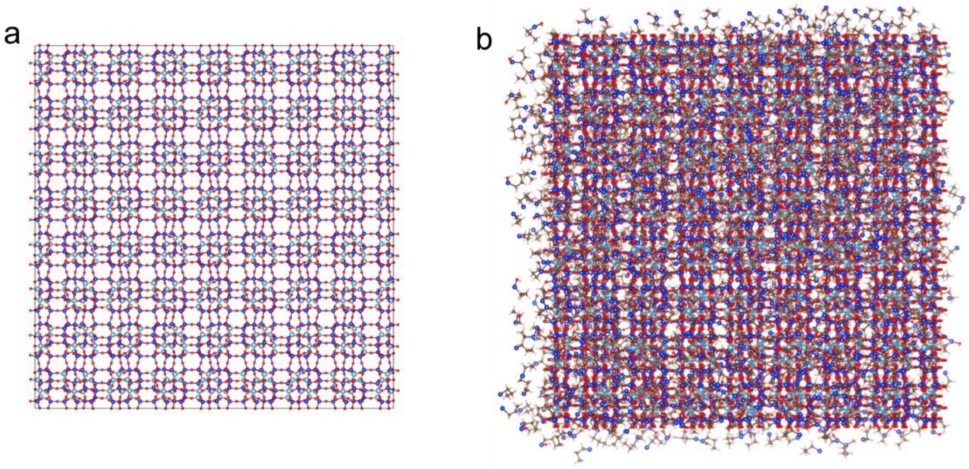

Supplementary Figure 18 The structures of (a) HieTS-1 and (b) HieTS-1-C_3.

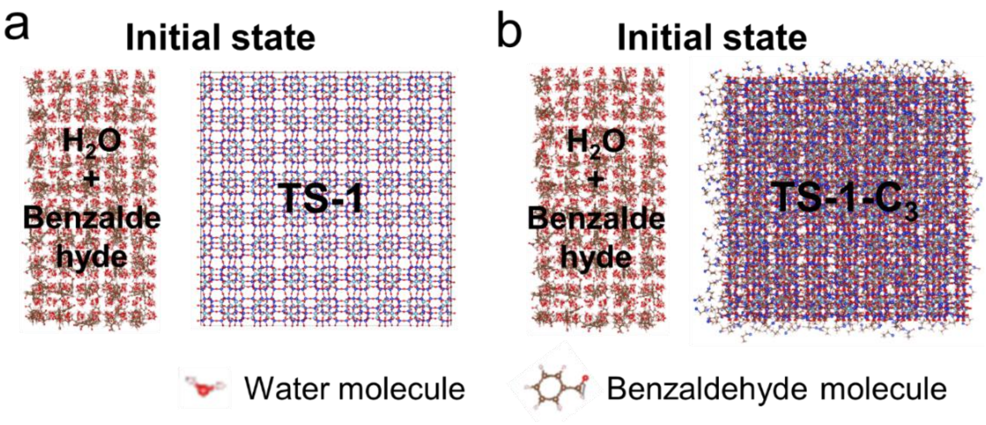

Supplementary Figure 19 The snapshot of initial state in the benzaldehyde diffusion simulation of (a) HieTS-1 and (b) HieTS-1-C_3.

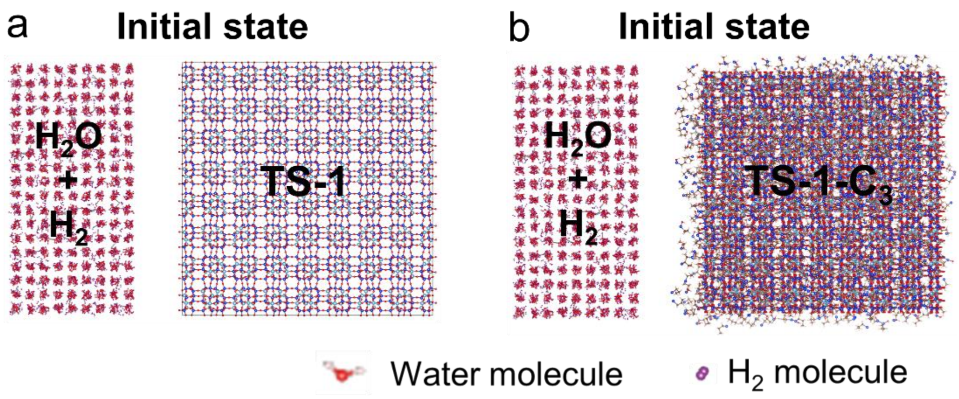

Supplementary Figure 20 The snapshot of initial state in the H_2 diffusion simulation of (a) HieTS-1 and (b) HieTS-1-C_3.

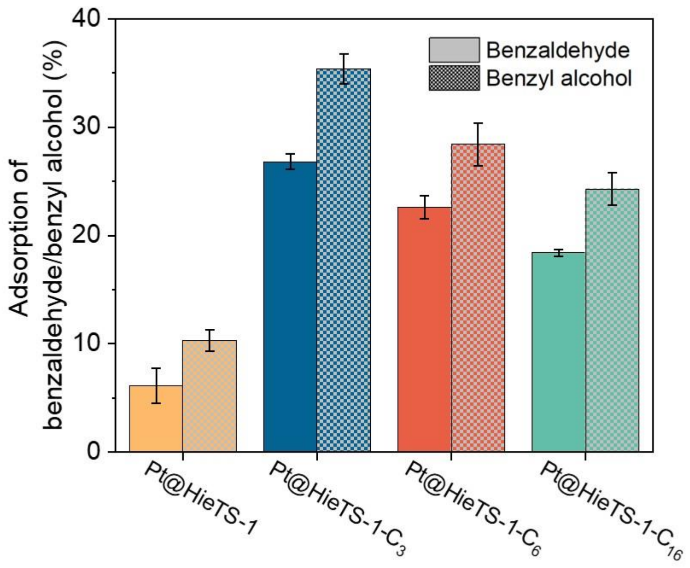

Supplementary Figure 21 Adsorption capacity of benzaldehyde (or benzyl alcohol) over different catalysts. Adsorption conditions: benzaldehyde (or benzyl alcohol) (0.47 mmol), catalyst (20 mg), water (5 mL). The carbon balance of the adsorption experiments approximated 95~97%. For all adsorption capacity, data points indicate the group means and error bars represent the group standard deviation.

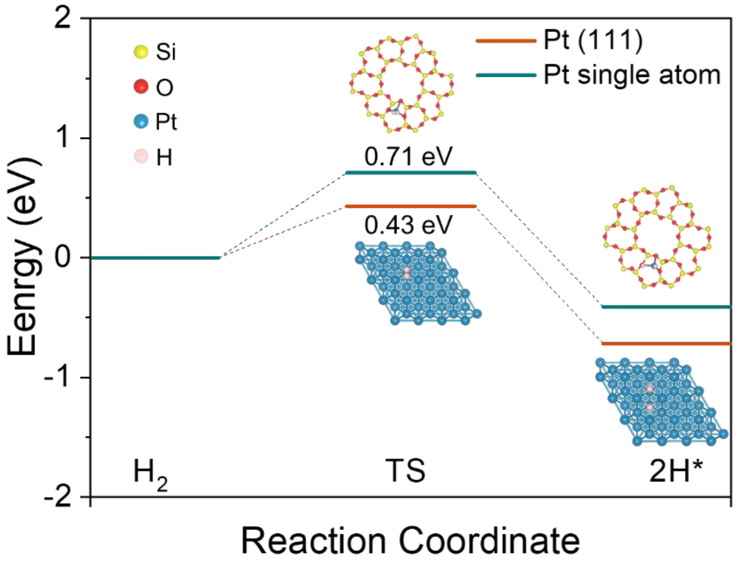

Supplementary Figure 22 Relative energy plots of H_2 dissociation on metallic Pt (111) surface and atomically dispersed Pt species surfaces.

DFT calculation was employed to comprehend the energy barrier for dihydrogen activation on Pt NPs and atomically dispersed $Pt^{\delta+}$ species (Supplementary Figure 22). the dissociation H_2 follows a heterolytic pathway on atomically dispersed $Pt^{\delta+}$, while it manifests homolytic dissociation on the metallic Pt (111) surface. Furthermore, the barrier of H_2 dissociation on atomically dispersed $Pt^{\delta+}$ species (0.71 eV) is higher than that on metallic Pt (111) surface (0.43 eV). This implies that the primary pathway for dihydrogen activation is heterolysis dissociation on Pt NPs, while the heterolytic dissocation on atomically dispersed $Pt^{\delta+}$ acts as an assistant role.

Supplementary Table 1 Textural and structural characteristics of various catalysts.

Entry	Catalyst	Pt loading (wt %)	S_{BET} (m^2 g^{-1})	V_t (cm^3 g^{-1})	d_{BJH} (nm)
1	Pt@HieTS-1	0.70	391.4	0.64	8.9
2	Pt@HieTS-1-C_3	0.69	392.6	0.58	8.4
3	Pt@HieTS-1-C_6	0.69	382.3	0.55	7.4
4	Pt@HieTS-1-C_{16}	0.64	371.2	0.51	7.6

Supplementary Table 2 Structural parameters extracted from quantitative EXAFS curve-fitting.

Sample	Shell	CN^a	R(Å)^b	σ^2(Å^2)^c	ΔE_0(eV)^d	R factor
Pt foil	Pt-Pt	12	2.76	0.0042	5.7	0.002
PtO_2	Pt-O	6	1.91	0.0019	12.8	0.02
Pt@HieTS-1	Pt-O	3.3±0.4	2.01	0.006	10.1	0.01
	Pt-Pt	3.8±1.0	2.76	0.004	8.1	

^{a}*CN*, coordination number; ^{b}*R*, distance between absorber and backscatter atoms; $^c\sigma^2$, Debye-Waller factor to account for both thermal and structural disorders; $^d\Delta E_0$, inner potential correction; *R* factor indicates the goodness of the fit. S_0^2 was fixed to 0.783, according to the experimental EXAFS fit of Pt foil by fixing CN as the known crystallographic value.

Supplementary Table 3 Assignments of IR bands observed on organosilane modified catalysts.

Catalyst	IR band (cm^{-1})	Vibrational modes	Ref.
Pt@HieTS-1-C_3	2944	the stretching vibration of -CH groups	8, 9
	2852	the stretching vibration of -CH_2- groups	
	1382	bending vibration of -CH_3	
Pt@HieTS-1-C_6	1637	the framework of benzene ring	10, 11
	932	the framework of benzene ring	
Pt@HieTS-1-C_{16}	2918	the stretching vibration of hydrocarbons species	12-14
	2840	the stretching vibration of hydrocarbons species	
	1461	vibration of methylene	

Supplementary Table 4 Comparison of various catalysts for the hydrogenation of benzaldehyde to benzyl alcohol in liquid phase.

Catalyst	Solvent	Reaction conditions Temp. (°C)	H_2 (atm)	Time (h)	Substrate conv. (%)	Product select. (%)	Ref.
Pt/@-ZrO_2/SBA	H_2O	50	10	1	100	99	15
Pt/SBA-15	H_2O	50	10	2.5	100	99	15
Pt/ZrO	H_2O	50	10	2.5	29	99	15
Pd/@ZrO_2/AC	H_2O	40	7	0.5	100	98	16
Ni_1Fe_1	H_2O	100	10	4	93	100	17
Ag-Fe_3O_4@CMC	H_2O	100	40	24	95	-	18
0.2Pt/$MgAl_2O_4$	ethanol	60	10	4	100	99	19
1Pt/$MgAl_2O_4$	ethanol	60	10	4	100	99	20
Pt/15TS	ethanol	25	40	0.75	94	99	21
Pt/TiO_2	ethanol	25	40	0.75	85	99	21
Ni_1Fe_1	ethanol	100	10	4	100	100	17
MgCoMo HT	ethanol	110	10	5	93	100	22
Pd_{1+NPs}/TiO_2	ethanol	25	1	0.25	100	98	23
Pd/NGC	ethanol	30	5	1	99	42	24
Pd/AC	p-xylene	50	2	1	98	86	25
Ni−5ReO_x/TiO_2	dioxane	120	20	0.5	63	100	26
Pd_{SA}/G	n-octane	60	7	1	99	97	27
Pt_2/mpgC_3N_4	isopropanol	120	80	9	100	99	20
Ni/Al_2O_3-SiC	isopropanol	90	20	2	77	93	28
Cu-Pt@TMS	isopropanol	110	10	3	100	100	29
This work	H_2O	50	1	1	96	100	

Supplementary Table 5 Catalytic hydrogenation of benzaldehyde with different catalysts.[a]

Entry	Catalysts	Solvent	H_2 (atm)	Time (h)	Yield of benzyl alcohol (%)	Carbon balance (%)
1	Pt@HieTS-1	H_2O	1	8	94	97
2	Pt@HieTS-1-C_3	H_2O	1	2.5	99	99
3	Pt@HieTS-1-C_6	H_2O	1	3.5	99	97
4	Pt@HieTS-1-C_{16}	H_2O	1	4.5	98	96
5	Pt@HieTS-1	H_2O	10	0.5	22	97
6	Pt@HieTS-1-C_3	H_2O	1	0.5	26	99

a Reaction conditions: benzaldehyde (0.47 mmol), Pt dosage (2.1×10^{-3} mmol), solvent (5 mL), 50 °C.

Supplementary Table 6 Simulation data of the penetration ratio of benzaldehyde/H_2 molecules for TS-1 and TS-1-C_3.

Entry	Molecule	Zeolite	Simulation
1	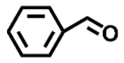	TS-1	24.2%
2		TS-1-C_3	72.3%
3	H_2	TS-1	23.3%
4		TS-1-C_3	75.8%

Supplementary References

[1] Thompson, A. P. et al. LAMMPS-a flexible simulation tool for particle-based materials modeling at the atomic, meso, and continuum scales. *Comput. Phys. Commun.*, **271**, 108171 (2022).

[2] Kokotailo, G. T. et al. Structure of synthetic zeolite ZSM-5. *Nature* **272**, 437–438 (1978).

[3] Huang, L. et al. Aldol condensation reaction in hierarchical ZSM-5 zeolite: A molecular dynamics simulation. *Microporous Mesoporous Mater.* **348**, 112393 (2023).

[4] Kresse, G. & Furthmüller, J. VASP the Guide (Universität Wien, Wien, Austria, 2007). Phys. Rev. B **47**, 558, (1993)

[5] Kresse, G. & Furthmüller, J. Efficiency of ab-initio total energy calculations for metals and semiconductors using a plane–wave basis set. *Comput. Mater. Sci.* **6**, 15–50 (1996).

[6] Kresse, G. & Furthmüller, J. Efficient iterative schemes for ab initio total-energy calculations using a plane-wave basis set. Phys. Rev. B **54**, 11169 (1996).

[7] Berendsen, H. J. C., Postma, J. P. M., Gunsteren, W. F. V., DiNola, A. & Haak, J. R. Molecular Dynamics with Coupling to an External Bath. *J. Chem. Phys.* **81**, 3684–3690 (1984).

[8] Jeon N, L. et al. Structure and stability of patterned self-assembled films of octadecyltrichlorosilane formed by contact printing. *Langmuir* **13**, 3382–3391 (1997).

[9] Zapata, P. A. et al. Hydrophobic zeolites for biofuel upgrading reactions at the liquid–liquid interface in water/oil emulsions. J. Am. Chem. Soc. 134, 8570–8578 (2012).

[10] Jin, Z. et al. Hydrophobic zeolite modification for in situ peroxide formation in methane oxidation to methanol. *Science* **367**, 193–197 (2020).

[11] Hoffmann H, Mayer U, Krischanitz A. Structure of alkylsiloxane monolayers on silicon surfaces investigated by external reflection infrared spectroscopy. *Langmuir* **11**, 1304–1312 (1995).

[12] Sugie, C. et al. Structure and thermodynamics of silicon oxycarbide polymer-derived ceramics with and without mixed–bonding. *Materials* **14**, 4075 (2021).

[13] Delaporte, N. et al. Alumina-flame retardant separators toward safe high voltage Li-Ion batteries. *J. Power Sources* **506**, 230189 (2021).

[14] Mirji, S. A. et al. Adsorption of octadecyltrichlorosilane on mesoporous SBA-15. *Appl. Surf. Sci.* **252**, 4097–4103 (2006).

[15] Zhang, Y., Zhou, J., Wang, F., Lv, M., & Li, K. Metal-metal oxide synergistic catalysis: Pt nanoparticles anchored on mono-layer dispersed ZrO_2 in SBA-15 for high efficiency selective hydrogenation. *J. Catal.* **421**, 12–19 (2023).

[16] Zhang, Y., Zhou, J., Li, K., & Lv, M. Synergistic catalysis of hybrid nano-structure Pd catalyst for highly efficient catalytic selective hydrogenation of benzaldehyde. *Catal. Today* **358**, 129–137 (2020).

[17] Wang, Y. et al. Facile synthesis of Ni/Fe_3O_4 derived from layered double hydroxides with high performance in the selective hydrogenation of benzaldehyde

and furfural. *Mol. Catal.* **528**, 112505 (2022).

[18] Li, A. Y., Kaushik, M., Li, C. J. & Moores, A. Microwave-assisted synthesis of magnetic carboxymethyl cellulose-embedded Ag-Fe_3O_4 nanocatalysts for selective carbonyl hydrogenation. *ACS Sustainable Chem. Eng.* **4**, 965–973 (2016).

[19] Yan, F. et al. Effect of the degree of dispersion of Pt over $MgAl_2O_4$ on the catalytic hydrogenation of benzaldehyde. *Chin. J. Catal.* **38**, 1613–1620 (2017).

[20] Tian, S. et al. Dual-atom Pt heterogeneous catalyst with excellent catalytic performances for the selective hydrogenation and epoxidation. *Nat. Commun.* **12**, 3181 (2021).

[21] Li, X. et al. Pt nanoparticles supported on highly dispersed TiO_2 coated on SBA-15 as an efficient and recyclable catalyst for liquid-phase hydrogenation. *J. Catal.* **300**, 9–19 (2013).

[22] Neethu, P. P., Venkatachalam, G., Venkatesha, N. J., Joseph, D. & Sakthivel, A. Cobalt-based hydrotalcite: a potential non-noble metal-based heterogeneous catalyst for selective hydrogenation of aromatic aldehydes. *Ind. Eng. Chem. Res.* **62**, 4976–4986 (2023).

[23] Kuai, L. et al. Titania supported synergistic palladium single atoms and nanoparticles for room temperature ketone and aldehydes hydrogenation. *Nat. Commun.* **11**, 48 (2020).

[24] Mironenko, R. M. et al. Liquid-phase hydrogenation of benzaldehyde over Pd-Ru/C catalysts: synergistic effect between supported metals. *Catal. Today* **278**, 2–9 (2017).

[25] Cattaneo, S. et al. Discovering the role of substrate in aldehyde hydrogenation. *J. Catal.* **399**, 162–169 (2021).

[26] Lin, W. et al. Surface synergetic effects of Ni-ReO_x for promoting the mild hydrogenation of furfural to tetrahydrofurfuryl alcohol. *ACS Catal.* **13**, 11256–11267 (2023).

[27] Yang, L. et al. Palladium single-atom catalysts synthesized by a gas-assisted redispersion strategy for efficient benzaldehyde hydrogenation. *Chem. Commun.* **59**, 5693-5696 (2023).

[28] Li, K., Jiao, Y., Yang, Z., & Zhang, J. A comparative study of Ni/Al_2O_3-SiC foam catalysts and powder catalysts for the liquid-phase hydrogenation of benzaldehyde. *J. Mater. Sci. Technol.* **35**, 159–167 (2019).

[29] Wang, S. et al. Ultrahigh Selective Hydrogenation of Furfural Enabled by Modularizing Hydrogen Dissociation and Substrate Activation. *ACS Catal.* **13**, 8720–8730 (2023).
